# Methicillin-Resistant *Staphylococcus aureus* Ocular Infection after Corneal Cross-Linking for Keratoconus: Potential Association with Atopic Dermatitis

**DOI:** 10.1155/2015/613273

**Published:** 2015-03-18

**Authors:** Romina Fasciani, Antonio Agresta, Alice Caristia, Luigi Mosca, Andrea Scupola, Aldo Caporossi

**Affiliations:** Department of Head and Neck Surgery, Ophthalmology Unit, Catholic University of the Sacred Heart “A. Gemelli”, Largo “A. Gemelli” 8, 00168 Rome, Italy

## Abstract

*Purpose*. To report the risk of methicillin-resistant *Staphylococcus aureus* (MRSA) ocular infection after UVA-riboflavin corneal collagen cross-linking in a patient with atopic dermatitis. *Methods*. A 22-year-old man, with bilateral evolutive keratoconus and atopic dermatitis, underwent UVA-riboflavin corneal cross-linking and presented with rapidly progressive corneal abscesses and cyclitis in the treated eye five days after surgery. The patient was admitted to the hospital and treated with broad-spectrum antimicrobic therapy. *Results*. The patient had positive cultures for MRSA, exhibiting a strong resistance to antibiotics. Antibiotic therapy was modified and targeted accordingly. The intravitreal reaction is extinguished, but severe damage of ocular structures was unavoidable. *Conclusion*. Riboflavin/UVA corneal cross-linking is considered a safe procedure and is extremely effective in halting keratoconus' progression. However, this procedure is not devoid of infectious complications, due to known risk factors and/or poor patients' hygiene compliance in the postoperative period. Atopic dermatitis is a common disease among patients with keratoconus and *Staphylococcus aureus* colonization is commonly found in patients with atopic dermatitis. Therefore, comorbidity with atopic dermatitis should be thoroughly assessed through clinical history before surgery. A clinical evaluation within three days after surgery and the imposition of strict personal hygiene rules are strongly recommended.

## 1. Introduction

Keratoconus is a progressive, ectatic corneal disease, causing stromal thinning and highly irregular astigmatism that may require a corneal graft to improve visual acuity in later stages [1]. The corneal cross-linking (CXL) procedure is based on the reaction between a riboflavin solution and UVA irradiation of corneal surface, leading to an increase of the mechanical strength of the corneal stromal tissue through the formation of covalent bonds between neighboring collagen molecules [2], definitively halting the progression of the disease.

Several clinical trials have reported that corneal cross-linking proved effective for the stabilization of keratoconus [3, 4]. The onset of stromal edema and transient corneal haze are typical postoperative findings, with a tendency to decrease and eventually disappear in the following months [4, 5]. The most frequently observed complications are represented by corneal scarring, sterile infiltrates [6–8], and delayed epithelial healing, whereas infectious keratitis, although rare, seems to be the most serious complication [9–16].

Keratoconus is commonly observed in patients with atopic disease, with itching causing repeated eye rubbing that may trigger or exacerbate corneal ectasia in genetically predisposed patients [17]. Moreover, a high incidence of* Staphylococcus aureus*, including MRSA variety, colonization of eyelid margins, and conjunctival sacs was observed in patients with atopic dermatitis [18, 19]. We report a case of severe ocular impairment due to MRSA infection occurring after UVA/riboflavin corneal cross-linking for keratoconus in a patient with atopic dermatitis.

## 2. Methods

A 22-year-old young man with bilateral evolutive keratoconus underwent UVA/riboflavin cross-linking in his right eye. A therapeutic contact lens (LAC) was applied in the treated eye at the end of the surgical procedure. Both anti-inflammatory and broad-spectrum antibiotic topical therapies were prescribed (ofloxacin 0,3% and indomethacin 0,1% ophthalmic solutions, one drop each every 6 hours).

A postoperative follow-up visit was scheduled for five days after surgery, in order to assess epithelial wound healing and to remove LAC.

**Figure 1:**
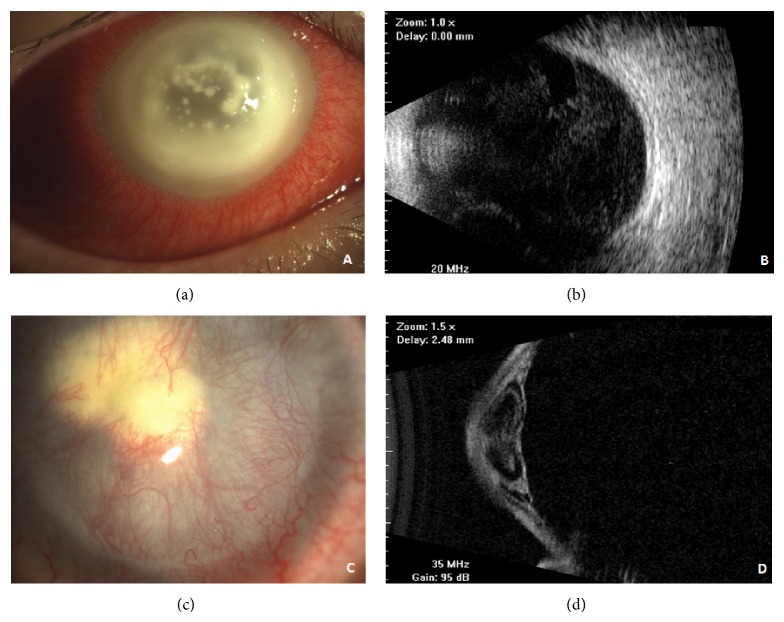
(a) Five days after CXL, slit-lamp examination revealed the presence of an annular corneal abscess, spared and scattered miliary infiltrates, and diffuse corneal edema with a strong inflammatory reaction in the ciliary body. The anterior chamber was not appreciable in detail. (b) Ultrasound B-scan showing vitreous reaction on postoperative day 7. (c) Slit-lamp image showing vascularized corneal pannus, with the presence of a dense leucoma in the center of the cornea. (d) Ultrastructural B-scan (UBM) evaluation showing the anteriorization of iris and lens planes, with the presence of angular synechiae and the absence of anterior chamber (2nd month after CXL).

On postoperative day 2, the patient presented with redness and purulent discharge, documented with the use of his personal smartphone camera, but he resolved to return for a follow-up visit only on postoperative day 5, as previously scheduled. At postoperative follow-up evaluation, the patient presented with periorbital and eyelid edema, corneal opacity, purulent discharge, and pain. Slit lamp examination documented the presence of annular corneal abscess, located just below the edges of the area of treatment, and of spared and scattered miliary infiltrates and diffuse corneal edema, with a strong inflammatory reaction occurring in the ciliary body and subsequent obfuscation of the anterior chamber (Figure 1(a)). Visual acuity was light perception with low ocular pressure and ultrasonography evaluation of the affected eye showed no signs of vitreous involvement.

Anamnestic questionnaire revealed the presence of atopic dermatitis treated with topical steroid ointment. Microbiological samples were quickly collected from eye secretions, LAC, and lid margin and bacterial cultures were obtained.

## 3. Results

At first postoperative visit, the patient was immediately hospitalized. A topical broad-spectrum antibiotic therapy was administered: tobramycin and chloramphenicol/tetracycline eye ointments qid, and moxifloxacin 0.5% eye drops every 4 hours, amikacin sulfate 0.5%, and amphotericin 0.12% eye drops qid were also prescribed. Atropine eye drops qid were also prescribed. In addition, an infectious disease specialist consultation was immediately requested and systemic therapy was prescribed as follows: oral fluconazole (200 mg bid), intravenous ceftazidime (1 gr bid), and intravenous vancomycin (500 mg qid).

On the following day, a progressive worsening of both ocular inflammation and corneal infection was observed, with the presence of a centrally located abscess and massive intracameral exudation, preventing visualization of intraocular structures. Both topical and systemic therapies remained unchanged while microbiological results were pending. Two days later, an eye ultrasound examination was performed documenting the onset of a vitreous reaction and the development of endophthalmitis was clinically suspected (Figure 1(b)). The patient's bacterial cultures were positive for methicillin-resistant* Staphylococcus aureus* (MRSA). Afterwards, the antibiogram showed a strong MRSA resistance to penicillin, moxifloxacin, gentamicin, ciprofloxacin, tobramycin, and oxacillin, with reduced sensitivity to vancomycin and linezolid, and a higher sensitivity to daptomycin, tetracycline, teicoplanin, and clindamycin.

Subconjunctival and intravitreal injections of vancomycin 1% were performed according to the protocols reported in literature for endophthalmitis [20–23]. No vitreous specimens were obtained considering that the etiologic agent was already known and that waiting for further microbiological results was impracticable due to the severity of infection. In addition, vitrectomy was not feasible because of unsatisfactory visualization of intraocular structures due to corneal opacities. On postoperative day 8, vancomycin 5% ophthalmic solution qid was started, whereas tobramycin ointment and moxifloxacin, amikacin sulfate, and amphotericin eye drops were discontinued. Systemic therapy was rapidly shifted to 1-hour intravenous infusions of linezolid 600 mg twice a day and daptomycin for intravenous use (4 mg/kg in 50 mL, 30-minute infusion) was subsequently added. After ten days of targeted therapy, resolution of corneal infection was confirmed by additional microbiological sampling revealing no bacterial growth. Nonetheless, severe corneal damage was observed, with a large trophic epithelial ulcer, calcium and/or drug deposition, partial stromal melting, and complete stromal opacification. Direct visualization of the anterior segment and vitreous chamber was not feasible, but B-scan ultrasonography performed on a daily basis showed a progressive reduction of vitritis. Systemic antibiotic therapy was immediately discontinued and eye drops were gradually tapered within seven days. Anterior segment optical coherence tomography (OCT) was performed, showing severe stromal thickening with high hyperreflective deposition obscuring posterior structures. In order to improve corneal epithelialization, a human amniotic membrane (HAM) graft was performed on the ocular surface using a single scleral 10/0 nylon running suture. One month later, HAM dissolved, sutures were removed, and a complete vascularized corneal pannus with stromal opacification was observed, despite resolution of the epithelial ulcer. Intraocular pressure measured by Schiötz tonometer was 18 mmHg. Two months after the onset of MRSA corneal abscess, the vascularized corneal pannus was still evident with the presence of a dense leucoma in the central cornea (Figure 1(c)). Anterior segment OCT was again performed revealing central stromal thinning with persistence of hyperreflective intrastromal deposition. Ultrastructural B-scan (UBM) evaluation (Figure 1(d)) showed a severe anteriorization of both iris and lens planes with the presence of angular synechiae and the absence of the anterior chamber. Six months after surgery, in consideration of a visual acuity of hand motion, combined with elevated intraocular pressure and severe damage to the anterior segment, further surgery was required (subtotal vitrectomy, anterior synechiolysis, iridectomy, penetrating keratoplasty with open-sky extracapsular lens extraction, and sclerally fixated IOL implant) with no improvement of final visual acuity.

## 4. Discussion

Riboflavin-UVA corneal cross-linking is a relatively safe procedure, with low complications rates [3, 4] and infectious keratitis as its most serious complication [9, 11, 12, 14, 15, 24, 25].

New lineages of MRSA, defined as community acquired MRSA (CA-MRSA), have emerged over the years with a propensity to cause infections, especially of the skin, in young individuals who have never been hospitalized [26, 27]. Moreover, a high incidence of* Staphylococcus aureus*, including MRSA variety, colonization of the eyelid margins, and conjunctival sacs, was reported among patients with atopic dermatitis. In particular,* Staphylococcus aureus* was isolated from 67% of patients suffering from atopic dermatitis versus only 6% of nonatopic control patients [28] and a high incidence of MRSA infections after scleral buckling procedures for retinal detachments has been reported in association with atopic dermatitis [29]. Furthermore, scattered cases of MRSA keratitis were described in case of keratoconus among patients with atopic dermatitis [19, 30]. These findings indicate the massive colonization of atopic skin by* Staphylococcus aureus* species due to a variety of functional abnormalities of the immune system [18], such as impaired IgA secretion in tears and low interferon-*γ* (IFN-*γ*) release from T-cells [31, 32], exacerbated by long-term oral/topical steroid therapy. Moreover, repeated eye rubbing due to the intense itching, typical of this condition, and bacterial colonization of nails are considered key factors in the dissemination of bacteria to body organs without eczematous lesions [33].

It should be noted that about 35% of patients with keratoconus are atopic [34] and about 14–30% have eczema [35–38]. Therefore, keratoconic patients could be more susceptible to developing MRSA keratitis. Surgical procedures involving disepithelization and the use of contact lenses could enhance the infectious risk in susceptible patients. Interestingly, as previously reported in the infectious keratitis survey promoted by ASCRS, evaluating trends of infectious keratitis following keratorefractive procedures, the most common organism cultured was MRSA, with an incidence of 1 infection in every 1102 procedures [39]. However, despite the high rate of association between keratoconus and atopic dermatitis, a review of existing literature showed few cases of MRSA infection following UVA/CXL [14, 16, 30]. This finding should be related to the sterilizing effect of UVA irradiation used during CXL procedure on the central cornea. In fact, CXL is also used for the treatment of resistant corneal infections, including MRSA variety [40, 41].

At the onset of corneal infection, our case presented with an annular stromal infiltrate surrounding the corneal limbus, just below the margin of the irradiated area, sparing the center of the cornea. Such a peculiar presentation in the earlier phase of keratitis could be related to UVA sterilizing action, which was effective only in the central corneal area, where the UVA irradiation spot was focused during the CXL procedure. To the best of our knowledge, our case represents the most severe MRSA corneal infection after UVA/riboflavin corneal cross-linking, complicated with vitritis. The severe evolution of the infection was probably due to delayed specialist referral and subsequent late diagnosis and delayed targeted treatment instauration, combined with strong antibiotic resistance.

Several newly discovered strains of MRSA have been described in literature, with a wide range of antibiotic resistance including vancomycin and newer drugs, such as linezolid and daptomycin, usually effective against MRSA species [42, 43]. Resistance to moxifloxacin seems to be intrinsic in antibiotic rather than being a new resistance stemming from mutations [44]. In our case, MRSA strain presented a low sensitivity to vancomycin and linezolid and a higher sensitivity to daptomycin, tetracycline, teicoplanin, and clindamycin. Since tetracycline topical ointment had already been administered and topic teicoplanin and clindamycin antibiotics were not yet available, intravenous injections of daptomycin were started. Intravitreal injections of daptomycin were not performed, as the local ethics committee denied authorizations for such procedure. Nevertheless, an intravitreal injection of vancomycin 1% was performed due to the clinical suspect of endophthalmitis and for medicolegal reasons. A decrease in vitreous reaction was observed in the following days, suggesting either the absence of an infectious endophthalmitis or the efficacy of daptomycin systemic therapy.

Despite resolution of MRSA ocular infection, the patient presented with severely impaired visual acuity and with severe damage to the anterior segment, requiring further surgeries in order to reduce intraocular pressure and preserve residual visual function.

To avoid the onset of postoperative infectious complications, we recommend a thorough preoperative anamnestic evaluation in order to determine the presence of atopic dermatitis. Povidone-iodine solution is the most rapid bactericidal antiseptic available against both MRSA and methicillin-sensitive* S. aureus* [45]. The most effective and reasonable regimen seems to be the application of 10% povidone-iodine for at least 5 minutes before surgery [46–48]. However, in case of elevated bacterial load, frequent among patients with atopic dermatitis [28], 13% of bacterial isolates remained viable, even with a long exposure time [47]. Therefore, prophylaxis with 1.25% povidone-iodine ophthalmic solution, one drop four times daily, in the affected eye constitutes a safe and effective precaution [49] that appears mandatory in all patients suffering from atopic dermatitis. Polymyxin B and trimethoprim may prove helpful for postsurgical MRSA prophylaxis [50].

Even a relatively safe procedure as CXL for keratoconus can be burdened by severe complications. When assessing the patient's operative risk, the ophthalmologist needs to consider the massive colonization of* Staphylococcus aureus* species on atopic skin and preoperative skin lesions swabs. A postoperative clinical evaluation within three days after surgery is mandatory in these cases and clinicians should carefully assess patient's compliance to postoperative instructions.
